# Rare complication of endoscopic band ligation for colonic diverticular bleeding

**DOI:** 10.1002/jgh3.12407

**Published:** 2020-08-08

**Authors:** Naoyuki Tominaga, Shinichi Ogata, Motohiro Esaki

**Affiliations:** ^1^ Department of Gastroenterology Saga‐Ken Medical Centre Koseikan, Japan Saga Japan; ^2^ Division of Gastroenterology, Department of Internal Medicine, Faculty of Medicine Saga University Saga Japan

**Keywords:** computed X‐ray tomography, diverticular diseases, endoscopic hemostasis, endoscopy, intestinal perforation

## Abstract

A 79‐year‐old female patient receiving maintenance hemodialysis was referred to our hospital because of massive hematochezia. Abdominal enhanced computed tomography (CT) demonstrated extravasation of contrast medium in the descending colon. We then performed urgent colonoscopy, and successful endoscopic hemostasis was achieved using endoscopic band ligation (EBL) for a bleeding colonic diverticulum. However, the patient unexpectedly complained of severe abdominal pain and fever 5 days after EBL, and abdominal CT revealed free air and mesenteric panniculitis. Emergency surgery was performed, and delayed colonic perforation at the EBL site was confirmed. Although rare, delayed perforation after EBL for colonic diverticular bleeding should be considered.

## Graphical abstract

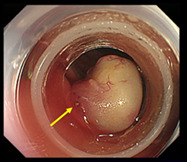

Endoscopic band ligation is a useful hemostatic technique for colonic diverticular bleeding. However, the possible risk of delayed colonic perforation should be considered.

## Introduction

Colonic diverticular bleeding (CDB) has been the most common cause of lower gastrointestinal bleeding.^1^ Endoscopic band ligation (EBL) has been used to achieve hemostasis in patients with CDB, and several reports suggest that EBL might be superior to endoscopic clipping (EC) for hemostasis.^2^, ^3^ The potential risk of perforation should be considered in EBL application,^4^ considering the thin intestinal walls of the diverticula.^5^ Here, we report a case of delayed perforation after CDB that was treated by EBL during hemodialysis.

## Case report

**Figure 1:**
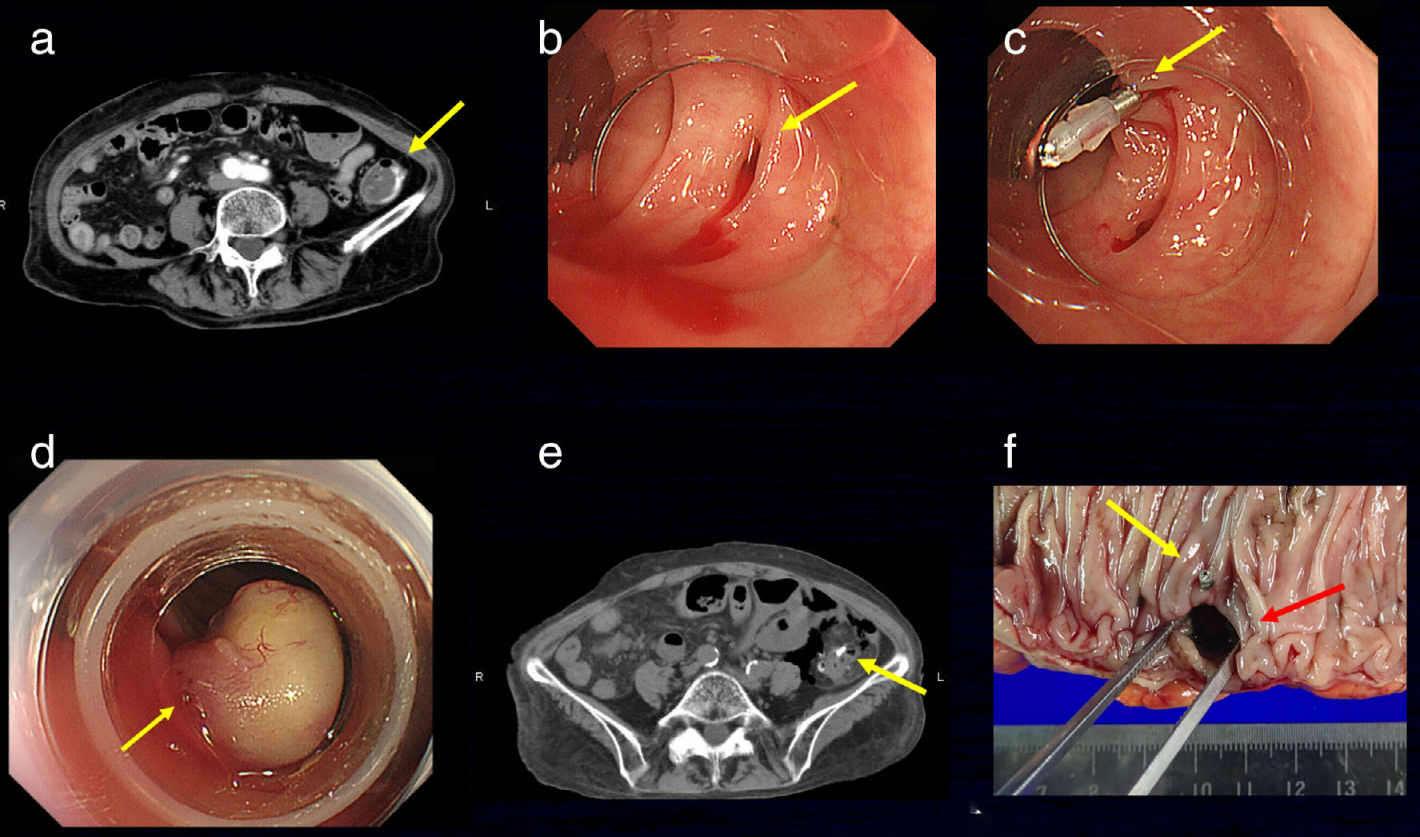
(a) Enhanced computed tomography demonstrating extravasation in the lower descending colon (arrow). (b) Bleeding is seen from the diverticulum (arrow). (c) The arrow shows the marking clip placed near the bleeding diverticulum. (d) The inverted diverticulum with exposed vessel (arrow) is ligated using endoscopic band ligation (EBL). (e) Emergency computed tomography demonstrating free air and mesenteric panniculitis in the descending colon around the marking clip (arrow). (f) A colonic wall defect is macroscopically visible at the EBL site (red arrow) near the marking clip (yellow arrow).

A 79‐year‐old Japanese woman who had been receiving maintenance hemodialysis was referred to our hospital for the treatment of massive hematochezia. Blood laboratory examination revealed decreased hemoglobin (76 g/L), and abdominal enhanced computed tomography (CT) demonstrated extravasation in the lower descending colon (Fig. 1a). After bowel preparation with polyethylene glycol solution, we performed colonoscopy and detected active CDB (Fig. 1b). EBL was selected for hemostasis for the CDB. We first placed the marking clip near the bleeding diverticulum (Fig. 1c) and withdrew the colonoscope. After attaching the band ligation device to the tip of the colonoscope, we reinserted the scope to the target diverticulum and suctioned the target diverticulum so that it was sufficiently inverted into the translucent hood of the band ligation device. The elastic band attached to the device was then released to ligate the inverted bleeding diverticulum (Fig. 1d). EBL was performed successfully without complications within 30 min.

On hospital day 5, severe abdominal pain and fever occurred unexpectedly. Abdominal CT demonstrated free air and mesenteric panniculitis in the lower descending colon (Fig. 1e), and emergent abdominal surgery was subsequently performed. Defects in the entire colonic wall at the site of the previous EBL were identified macroscopically (Fig. 1f), and histological examination revealed ulcers with neutrophils and fibrin deposits in the large intestinal mucosa.

## Discussion

CDB is the most common cause of lower gastrointestinal bleeding, and techniques such as epinephrine injection, thermal coagulation, hemoclipping, and EBL have been used for endoscopic hemostasis. Among these techniques, EBL has been used increasingly as a hemostatic technique for CDB in Japan because early (within 30 days) and late (within 1 year) rebleeding rates were significantly lower in CDB patients treated by EBL compared with those undergoing hemoclipping.^6^, ^7^, ^8^, ^9^

Two theories explain the hemostatic mechanisms of EBL for CDB, one being that ligating superficial blood vessels with EBL blocks the blood flow in the afferent vessels of the diverticulum and the other being submucosal fibrosis of the diverticulum, as well as ligation of the bleeding vessels. However, the potential risk of colonic perforation should be noted because of the lack of a proper muscular layer in colonic pseudodiverticula.

There have been two reported cases involving complicated colonic perforation after EBL for CDB.^4^, ^10^ One patient was treated with long‐term prednisolone (>10 mg/day) and aspirin (100 mg/day) for Takayasu arteritis, suggesting the contribution of delayed wound healing to the complication. However, although we found no underlying condition in the latter case or in our patient, all colonic diverticula subsequently developing perforation after EBL for CDB were located in the left colon.

Generally, the muscularis propria does not prolapse following the standard EBL procedure. However, considerable anatomical variation and curvature can be found in the left colon, especially the sigmoid colon. In this situation, it seems plausible that excessive suction on the colonic wall during EBL can prolapse the entire colonic diverticula and surrounding colonic wall, leading to subsequent impaired tissue blood flow and colonic perforation. Therefore, the possible risk of delayed colonic perforation should be considered when performing EBL for CDB.
